# The relationship between non-communicable disease risk and mental wellbeing in adolescence: a cross-sectional study utilising objective measures in Indonesia

**DOI:** 10.1186/s12889-024-20902-1

**Published:** 2024-12-18

**Authors:** Karly I. Cini, Dorothea Dumuid, Kate L. Francis, Nisaa R. Wulan, Susan M. Sawyer, Fransisca Handy Agung, Minh D. Pham, Elissa C. Kennedy, Jane Fisher, Thach Tran, Bernie E. Medise, Yoga Devaera, Aida Riyanti, Budi Wiweko, Fransiska Kaligis, Tjhin Wiguna, Ansariadi Ansariadi, Peter S. Azzopardi

**Affiliations:** 1 Centre for Adolescent Health, Murdoch Children’s Research Institute, Melbourne, Australia; 2 Department of Paediatrics, School of Medicine Dentistry and Health Sciences, University of Melbourne, Melbourne, Australia; 3 Burnet Institute, Melbourne, Australia; 4 Alliance for Research in Exercise, Nutrition and Activity (ARENA), Allied Health & Human Performance, University of South Australia, Adelaide, Australia; 5 Faculty of Medicine, Universitas Pelita Harapan, Tangerang, Indonesia; 6 School of Public Health and Preventive Medicine, Monash University, Melbourne, Australia; 7 Cipto Mangunkusumo Hospital, Jakarta, Indonesia; 8 Department of Child Health, Faculty of Medicine, Universitas Indonesia, Jakarta, Indonesia; 9 Universitas Indonesia Hospital, Depok, Indonesia; 10 Department of Obstetrics and Gynaecology, Faculty of Medicine, Universitas Indonesia, Jakarta, Indonesia; 11 Indonesia Medical Education Research Insitute (IMERI), Faculty of Medicine, Universitas Indonesia, Jakarta, Indonesia; 12 Department of Psychiatry, Faculty of Medicine, Universitas Indonesia, Jakarta, Indonesia; 13 Centre for Epidemiology and Population Health Studies, Faculty of Public Health, Hasanuddin University, Makassar, Indonesia; 14 Adolescent Health and Wellbeing Program, The Kids Research Institute, Adelaide, Australia

**Keywords:** Adolescent, Mental health, Non-communicable diseases, Risk factors, Indonesia, Wellbeing, Quality of life

## Abstract

**Background:**

Risk factors for non-communicable diseases (NCDs, cardiovascular diseases, cancers, chronic respiratory diseases, diabetes, and mental disorders) arise in adolescence but are mostly framed as relevant to health in adulthood; little is known about the relationship between co-occurring NCD risks and mental wellbeing in young people. This study aims to describe the prevalence and co-occurrence of distinct NCD risk factors, and how they relate to current mental wellbeing amongst adolescents in Indonesia, a young and populous country where NCD burden is increasing rapidly.

**Methods:**

We assessed NCD risk and mental wellbeing amongst 1,331 school-based 16–18-year-olds in Jakarta (*N* = 609) and South Sulawesi (*N* = 722). Five domains of NCD risk (adiposity, substance use, physical inactivity, excess sedentary time, and diet) were either measured or self-reported. In Jakarta, we also measured blood glucose, triglycerides, cholesterol, and blood pressure. Wellbeing was assessed using three indicators: general quality of life (QoL), physical function QoL, and psychological distress. We used linear regression to estimate the associations between co-occurring risks and wellbeing, adjusted for covariates of wellbeing: province, sex, socioeconomic status, and religion.

**Results:**

NCD risk clustering was common, and more than half of adolescents had co-occurring risks in 3 or more of the 5 domains (58.9% (95%CI 53.7—63.9)). Adolescents with any NCD risk were more likely to report psychological distress, with this relationship most pronounced in those with excess sedentary time spent on video gaming and computer use. A higher number of NCD risk factors was associated with poorer psychological wellbeing and decreased general and physical function QoL. In the Jakarta subsample, reduced HDL and raised blood glucose was associated with psychological distress; and a higher number of risk biomarkers was associated with lower physical function QoL.

**Conclusions:**

Our analysis also shows that these NCD risks (both individual risks and co-occurring risk count) are related to poorer profiles of mental wellbeing in adolescents, after adjusting for likely confounders.

**Supplementary Information:**

The online version contains supplementary material available at 10.1186/s12889-024-20902-1.

## Background

Non-communicable diseases (NCDs), such as cancer, cardiovascular disease, diabetes, chronic respiratory disease and mental disorders are now the leading cause (70%) of disease burden globally [1, 2]. Long considered an issue for high-income settings, we now know that the majority (75%) of all NCD-related deaths and 85% of premature deaths (those in people aged 30–69 years) occur in LMICs [1]. In Indonesia NCDs were the cause of over 42 million deaths in 2019, more than 74% of all deaths, many of which are largely preventable [1, 2]. NCDs have common behavioural risks (tobacco use, harmful use of alcohol, physical inactivity, poor diet, and air pollution) and biomarkers of risk (obesity, raised blood pressure, increased blood sugar levels, and adverse blood lipid profile) [3], many of which, if identified, can be modified to reduce the risk of disease. Evidence from Indonesia indicates that NCDs and their risk factors are rapidly increasing and differ significantly by socio-demographic characteristics like sex, province, and age, highlighting the urgency for preventative action [4–6].

While adults bear a higher burden of death and disability from NCDs, adolescence is when many NCD risk factors arise and represents an important period for preventative intervention [7, 8]. Indeed, as many NCDs are chronic and incurable, prevention plays a dominant role in disease control, and it is now well established that prevention should have a life course approach [7, 9, 10]. However, a challenge to this approach is that preventative intervention targeting adolescence is largely framed with benefits later in the life-course; this is at odds with adolescent neurodevelopment where interventions that bring immediate impacts to young people and their wellbeing are more likely to be effective [8]. Beyond reducing any future NCD burden, early investment in NCD prevention may have the potential to improve the immediate quality of life and wellbeing of adolescents.

There is increasing evidence that NCD risks are highly prevalent during adolescence [11, 12]. Some studies from high income settings show that these individual risk factors (such as lack of physical activity, sedentary behaviour, soft drink consumption, and air pollution exposure) have a negative impact on current adolescent mental health [13–15] and health-related quality of life (QoL) [16]. This evidence is yet to be replicated in low- or middle-income settings. Further interrogation is needed into how co-occurring risk factors (experiencing multiple risk factors at once) can impact adolescents’ current wellbeing and quality of life. This is of particular importance in low-resource countries, such as Indonesia, while they begin to consider potential public policy strategies to mitigate the impact of rapidly rising NCDs.

## Methods

This study aims to describe the prevalence, sociodemographic distribution, and co-occurrence of NCD risk factors in Indonesian adolescents living in Jakarta and South Sulawesi. We also aim to understand how the presence of NCD risk factors is associated with the current wellbeing and quality of life of adolescents. For Jakarta, we describe the prevalence and distribution of cardiometabolic biomarkers (raised blood pressure, raised glycated haemoglobin (HbA1c), raised triglycerides, reduced High-density Lipoprotein (HDL) cholesterol). Furthermore, we explore whether there is an association between adolescents’ current wellbeing and quality of life and these risk biomarkers. This cross-sectional study involved a representative school-based survey, anthropometric assessment, and a sub-sample with biomarker assessment. The broader project was a cross-country collaboration between Indonesia and Australia and investigators included researchers and clinicians from Jakarta, Makassar, and Melbourne. Given the sensitive nature of the study, we prioritised community engagement, with community forums held early in the development of the study and again prior to data collection. Further information on study method and design are fully detailed elsewhere [17].

### Study setting and population

The study was conducted in two provinces of Indonesia, Jakarta (highly urbanised), and South Sulawesi (urban, peri-urban, and rural), selected for their differing geographic and socio-economic factors [17]. The study population was adolescents aged 16–18 years who had attended school in the past 90 days.

### Sampling, selection criteria, and recruitment

There is further detail in the published protocol on the sampling, selection criteria and recruitment for this mixed-methods study, briefly, we report details relevant to this analysis here [17]. We used a multi-stage sampling method, randomly selecting 12 schools and then three classes (from grade 10, 11, and 12) to participate from each province. All students in selected classes were invited to participate if they met the selection criteria. Consent information was sent home to parents or guardians with 2,509 school students and 1,337 (53.2%) returned signed consent forms; 611 (48.3%) from Jakarta and 726 (58.3%) from South Sulawesi. Along with written parental consent, it was a requirement of data collection that adolescent participants give informed assent to participate before beginning the survey and other data collection.

### Procedure

Data collection took place between February and April 2018. Students completed the survey on a tablet during a single class period and anthropometric measures in the following period. In Jakarta, biomarkers were also collected at this point and participants were seated and resting for collection. The process took about 2 h for an individual and each school completed data collection in one day.

#### Anthropometry

Participants were weighed once using Seca 877 digital scales, with measurements recorded to the nearest 100 g. Standing height was measured using a rigid stadiometer (Shorrboard portable). Two readings were taken, if measures differed by > 0.5 cm a third was taken. Waist circumference was measured using a SECA 201 constant tension tape. Two readings were taken, if measures differed by > 1.0 cm a third was taken.

#### Biomarkers

Serum biomarkers included triglycerides, high-density lipoprotein (HDL) cholesterol, and glycated haemoglobin A1C (HBA1c). Venous blood was collected by a trained phlebotomist who observed universal precautions in the first-aid area of each school, participants were not fasting. Blood pressure was measured using an automated wrist sphygmomanometer (Omron HEM-6121); two measurements were taken at one-minute intervals, and if the systolic reading differed by > 10 mmHg or the diastolic by > 6 mmHg, a third measurement was taken. Biomarkers were collected in Jakarta only, due to logistical and ethical restraints around following up any clinically significant results [17]. Biomarkers were considered clinically relevant and participants who met criteria of concern were followed up with a letter to their parent or guardian with information about the result that contained advice to consult with their local doctor [17].

### Measures

The 10-item Kessler Psychological Distress Scale (K10) was used to measure general psychological distress. A two-part validation study was undertaken, the K10 was adapted to Bahasa Indonesia through a translation and cultural validation process and then formally validated with a subsample of psychiatrist-administered interviews using the Mini International Neuropsychiatric Interview for Children and Adolescents (MINI-Kid) [18].

General quality of life was measured using the 15-item Youth Quality of Life Instrument-Short Form (YQoL-SF). Health-related quality of life for physical function was measured with the 8-item Pediatric Quality of Life Inventory (PedsQL) Physical function scale. For a detailed description of the outcomes and predictor variables used in this study including the operational definitions, see Table 1.

**Table 1:** Description of measures

	**Name of measure, source**	**Description**	**Definition and interpretation**
**Wellbeing Outcomes**	**Psychological Distress.** Kessler 10 (K10) [19]	10 items. Measure of psychological distress amongst adolescents, assessing symptoms over the past 4 weeks	The range for the K10 is 0–40. The summary score used is a total of all 10 items. A higher score indicates greater psychological distress
	**General Quality of Life.**Youth Quality of Life Instrument-Short Form (YQoL-SF) [20].	15 items. Multidimensional tool that asses general quality of life of adolescents aged 11 to 18 years. Responses to each item are on a ten-point Likert scale	YQoL-SF is recoded to a 100-point scale, a higher score indicates better quality of life. The summary score used is a mean of the recoded items
	**Health-related Quality of Life.**The Pediatric Quality of Life Inventory Physical function sub-scale (PedsQL-PF) [21].	8 items. A health-related quality of life scale which assesses physical ability and symptoms over preceding 30 days. Responses to each item are on a five-point Likert scale	The physical functioning sub-scale is recoded to a 100-point scale, a higher score indicates better quality of life. The summary score used is a mean of the recoded items
**Risk Factors:** All risk factors were dichotomised with 1 indicating risk definition met			
**Adiposity Domain**	High Body Mass Index (BMI)	2 items. Mean height and weight used to calculate BMI (weight in kilograms divided by height in meters squared). Age-sex-adjusted BMI Z-score computed using World Health Organization (WHO) 2007 reference chart [22, 23].	Risk was defined as BMI z-score > 1, which corresponds to the + 1 SD defined as overweight by WHO [23].
	High Waist Circumference	1 item. Mean waist circumference was calculated from the two nearest waist measurements	Risk was defined as waist circumference ≥ 90 cm for males; ≥ 80 cm for females [24].
**Substance use Domain**	Tobacco use. Adapted from Global Youth Tobacco Survey (GYTS) [25].	2 items. Questions relating to current cigarette smoking, and frequency of smoking	Risk was defined as smoking cigarettes at a frequency of weekly or more
	Alcohol use. Adapted from YRBSS [26].	2 items. Questions relating to lifetime and current frequency of alcohol use in the past 30 days	Risk was defined as at least one drink of alcohol in the past 30 days
**Physical inactivity Domain**	Lack of daily moderate to vigorous physical activity. Adapted from HBSC [27, 28].	1 item. Question on frequency and duration of physical activity over the past week. Equivalent to WHO PA guideline for 11–17-year-olds [29].	Risk was defined if an individual did not get 60 min of moderate to vigorous physical activity daily
	Lack of vigorous physical activity. Adapted from HBSC [27, 28].	1 item. Question on frequency of vigorous physical activity over the past month	Risk was defined if an individual did vigorous physical activity less than twice per week
**Sedentary Domain**	High** s**edentary activity. Adapted from HBSC [27, 28].	1 item. Questions relating to time spent watching TV (on average)	Risk was defined as watching TV more than 2 h per day
	High** s**edentary activity. Adapted from HBSC [27, 28].	1 item. Questions relating to time spent using a laptop and playing video games (on average)	Risk was defined as gaming/ computer use more than 2 h per day
**Unhealthy diet Domain**	Insufficient fruit and vegetable intake. Adapted from HBSC [27, 28].	2 items. Questions relating to weekly consumption of fruits, and of vegetables	Risk was defined as fruits or vegetables consumed less than daily
	Increased sweets or soft drink intake. Adapted from HBSC [27, 28]	2 items. Questions relating to weekly consumption of sweets, and of soft drinks	Risk was defined as sweets or soft drinks consumed at least daily
**Risk Count**	Co-occurring risk exposure: Count from 5 risk domains	A count of the risk domains that an individual had ≥ 1 risk/s present. For example, an individual with risk factors tobacco use, alcohol use, and high BMI would have a domain count of 2: substance use and adiposity	Range 0–5. A higher score indicates more co-occurring risk factors across multiple domains. The binary variable was defined as those with risk factors in ≥ 3 domains
**Biomarkers:**All risk factors were dichotomised with 1 indicating risk definition met. Biomarkers were selected, and risk cut points defined, based on the international diabetes federations’ criteria for metabolic syndrome in children and adolescents, and published in the study protocol [17, 24].			
**Risk Biomarkers**	Raised triglycerides	1 item. Biomarker assays analysed, triglyceride level reported	Risk defined as triglyceride level of ≥ 1.7 mmol/l [24].
	Reduced high-density lipoprotein (HDL) cholesterol	1 item. Biomarker assays analysed, HDL cholesterol level reported	Risk defined as HDL level of < 1.03 mmol/l in males, < 1.29 mmol/l in females [24].
	Raised blood pressure	2 items. Mean systolic and mean diastolic blood pressure	16-17yrs risk defined as systolic or diastolic blood pressure ≥ 90th centile for age, sex, height [30]. 18yrs, risk defined as systolic ≥ 130 mmHg or diastolic ≥ 85 mmHg [31].
	Raised glycated haemoglobin (HbA1c)	1 item. Biomarker assays analysed, HbA1c % reported	Risk defined as HbA1c ≥ 5.7% [24].
**Biomarker Risk Count**	Co-occurring biomarkers exposure: Count from 5 biomarkers	A count of 0–5 was assigned for the presence of high waist circumference, high triglycerides, low HDL, high blood pressure, and high blood sugar	Range 0–5. A higher score indicates the presence of biomarkers for risk. The binary variable was defined as those with ≥ 3 risk biomarkers

Socio-demographics included province (Jakarta or South Sulawesi), age (16, 17, or 18 years), sex (participants had the option to select Male, Female, or ‘other’ and specify their gender, grade at school (10, 11, or 12), self-report family socio-economic status (average to very well off, or below average to not at all well off), religious affiliation (Muslim or others (including Christianity, Buddhism, Hinduism, or none)), living situation (living with parents and stepparents or others (including grandparents, friends, dormitory house)).

### Data management and analysis

Unweighted descriptive statistics were calculated for the participant characteristics and wellbeing outcomes (psychological distress, YQoL, and PedsPF) using number and percentage for categorical variables and mean (standard deviation (SD)) for continuous variables.

Post stratification inverse probability weights were applied for the complex school-based survey design. Population weighted prevalence estimates and 95% confidence intervals were calculated for the dichotomised individual risk factors (self-report, anthropometry, and biomarkers) and co-occurring risk factors, and population weighted mean (SD) was calculated for the continuous co-occurring risk counts (risk factors and biomarkers).

We dichotomised socio-demographic factors: sex, province, family SES, living situation, and used disaggregated population weighted prevalence estimates to calculate risk ratios (RR) for each risk factor by socio-demographic factors. The relative risks and 95% confidence intervals were estimated using Poisson regression with robust error variance, and associations with p value < 0.05 were considered statistically significant.

Using a generalised linear-regression model we estimated the association between individual and co-occurring NCD risk factors and the wellbeing outcomes across the whole sample, and by males and females separately. Within the Jakarta subsample we estimated the association between individual and co-occurring risk biomarkers and wellbeing outcomes for Jakarta overall, and by males and females separately. We ran separate regression analyses for each wellbeing outcome and adjusted for the significant correlates: province, sex, family socioeconomic status, and religious affiliation. When the models were restricted to one sex only, the covariates included province, family socioeconomic status, and religious affiliation. Associations with p < 0.05 were considered statistically significant. All data cleaning and analyses were performed using STATA SE version 18.0.

#### Ethics

The study was approved by the Alfred Hospital Human Research Ethics Committee, Melbourne, Australia (approval number 114/17) and the Ethics Committee of the Faculty of Medicine, Universitas Indonesia (approval number 714/UN2.F1/ETIK/2017). All participants needed written informed consent from a parent or guardian, and to give their own informed assent, to participate in data collection.

#### Role of funding

The funder played no role in study design, data collection, data analysis, data interpretation, or writing of the paper. The authors had full access to the data presented in this manuscript and had responsibility for the decision to submit for publication.

## Results

### Participant characteristics

1,331 school-going adolescents took part in the current study, with 722 residing in South Sulawesi and 609 in Jakarta. From the original school sample of 1337 participants, 6 were excluded prior to analysis due to missing key demographic characteristics, sex and age. The median age of the sample was 17 years (Mean 16.7, SD 0.71) and females accounted for slightly more than half of the sample, 735 (55.2%) and no participants chose to specify their gender as ‘other’. Around half of the sample report their socio-economic status as below average. The main religious affiliation was Muslim (93.9%) and about 80% of our sample lived at home with their parents or stepparents (Table 2).

**Table 2:** Descriptive characteristics and mental wellbeing outcomes stratified by sex

	Female (*n* = 735)		Male (*n* = 596)		Total (*N* = 1331)	
	n	(%)	n	(%)	N	(%)
Province						
Jakarta	348	(47.3)	261	(43.8)	609	(45.8)
South Sulawesi	387	(52.7)	335	(56.2)	722	(54.2)
Age in years						
16	389	(52.9)	239	(40.1)	628	(47.2)
17	261	(35.5)	257	(43.1)	518	(38.9)
18	85	(11.6)	100	(16.8)	185	(13.9)
Grade at School						
Year 10	236	(32.1)	159	(26.7)	395	(29.7)
Year 11	262	(35.6)	234	(39.3)	496	(37.3)
Year 12	237	(32.2)	203	(34.1)	440	(33.1)
Family Socio-Economic Status, self-report						
SES Average or higher	342	(48.0)	282	(50.4)	624	(49.1)
SES Below average	370	(52.0)	277	(49.6)	647	(50.9)
*Missing*	*23*		*37*		*60*	
Living Situation						
Live with parents or stepparents	575	(80.4)	453	(77.8)	1,028	(79.3)
Live with others (grandparents, friends, others)	140	(19.6)	129	(22.2)	269	(20.7)
*Missing*	*20*		*14*		*34*	
Religion type						
Muslim	713	(97.1)	533	(89.9)	1,246	(93.9)
Christianity, Buddhism, Hinduism, or None	21	(2.9)	60	(10.1)	81	(6.1)
*Missing*	*1*		*3*		*4*	
**OUTCOMES, Mean (SD)**	M	(SD)	M	(SD)	M	(SD)
K10 Psychological Distress, Total 0–40	13.9	(7.2)	11.4	(6.5)	12.8	(7.0)
*Missing*	*52*		*63*		*115*	
PedsQL-Physical Functioning, Mean 0–100	74.2	(15.5)	81.2	(15.3)	77.3	(15.8)
Youth Quality of Life-Short Form, Mean 0–100	63.9	(19.6)	68.4	(20.8)	65.9	(20.3)

### Distribution of risk behaviours

#### Self-report risk factors

**Fig. 1:**
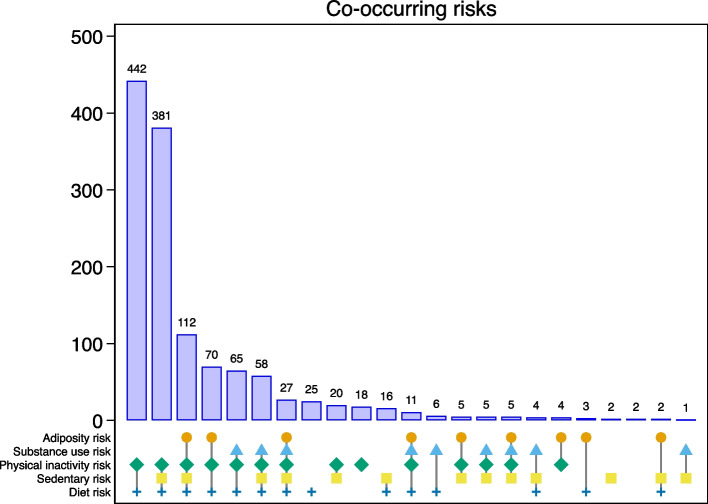
Risk factor co-occurrence across the full sample, by risk domain, sorted by frequency. Note: The groups are exclusive of each other, the icons indicate which risk domains adolescents were exposed to, and the number indicates the number of people in each group

The most common risk factors overall were physical inactivity (daily MVPA) and inadequate fruit and vegetable consumption (Table 3). Just over half of adolescents (58.9%, 95%CI 53.7 to 63.9) in our sample had risks in three or more domains, with a greater prevalence in males (65.4%, 95%CI 59.0 to 71.3) than females (53.6%, 95%CI 47.8 to 59.4), Table 3. Figure 1 is a visual representation of exclusive co-occurring risk factor clusters. The most common cluster was diet and physical inactivity; the most common cluster with three or more domains was diet, physical inactivity, and excess sedentary time, followed by diet, physical inactivity, excess sedentary time, and adiposity.

**Table 3:** Weighted estimates for NCD risk factors and biomarkers, by sex. Estimate is weighted population prevalence, unless otherwise noted

		Female			Male			Total		
Domain	Individual risk factor	n/N, estimate, (95%CI)			n/N, estimate, (95%CI)			n/N, estimate, (95%CI)		
Adiposity	High BMI	117/ 735	15.9%	(12.7,19.7)	117/ 596	19.7%	(14.4,26.3)	234/1331	17.6%	(14.0,21.8)
	High waist	96/ 735	13.0%	(9.1,18.2)	63/ 596	10.6%	(6.5,16.7)	159/1331	11.9%	(8.8,15.9)
Substance use	Smoking	6/ 625	1.0%	(0.5, 2.1)	141/ 436	32.3%	(23.6,42.5)	147/1061	13.9%	(8.6,21.7)
	Alcohol use	8/ 707	1.2%	(0.6, 2.5)	71/ 568	12.6%	(4.9,28.7)	80/1275	6.3%	(2.4,15.2)
Physical inactivity	Inadequate MVPA	707/ 735	96.1%	(94.8,97.2)	547/ 595	91.9%	(89.4,93.8)	1254/1330	94.2%	(93.0,95.2)
	Inadequate VPA	480/ 735	65.3%	(59.8,70.4)	207/ 595	34.7%	(30.2,39.5)	687/1330	51.6%	(46.3,56.9)
Sedentary	Excess TV	261/ 728	35.9%	(30.2,42.1)	179/ 590	30.3%	(24.7,36.5)	440/1318	33.4%	(28.7,38.4)
	Excess gaming	150/ 662	22.6%	(17.9,28.1)	210/ 546	38.4%	(31.2,46.1)	359/1209	29.7%	(24.7,35.3)
Diet	Lack of fruit or veg	670/ 735	91.1%	(88.3,93.3)	536/ 596	89.9%	(86.2,92.7)	1206/1331	90.6%	(88.3,92.4)
	Excess sweets/ soft drink	232/ 735	31.6%	(27.5,36.1)	152/ 596	25.5%	(21.4,30.1)	385/1331	28.9%	(25.6,32.4)
Co-occurring risk count (0–5)^a^		735	2.6 (0.72)	(2.5, 2.7)	596	2.9 (0.92)	(2.7, 3.1)	1331	2.7 (0.83)	(2.6, 2.9)
Co-occurring binary (≥ 3 risks)		394/ 735	53.6%	(47.8,59.4)	390/ 596	65.4%	(59.0,71.3)	784/1331	58.9%	(53.7,63.9)
Biomarkers (Jakarta only)										
	High waist circumference	74/ 374	19.7%	(12.7,29.4)	50/ 306	16.4%	(9.6,26.5)	124/ 680	18.2%	(12.4,25.8)
	Reduced HDL	194/ 284	68.2%	(55.5,78.7)	86/ 224	38.1%	(26.2,51.6)	279/ 509	54.9%	(46.4,63.1)
	Raised triglycerides	46/ 284	16.1%	(11.1,22.8)	62/ 224	27.7%	(19.9,37.1)	108/ 509	21.2%	(15.6,28.1)
	Raised blood glucose	11/ 284	3.9%	(1.3,11.6)	14/ 225	6.0%	(2.8,12.3)	25/ 510	4.8%	(2.1,10.6)
	Raised blood pressure	25/ 374	6.7%	(3.3,13.2)	39/ 303	12.7%	(7.1,21.7)	64/ 677	9.4%	(5.1,16.6)
Co-occurring risk count (0–5)^a^		284	1.1 (0.94)	(0.9, 1.4)	225	1.0 (1.02)	(0.8, 1.3)	510	1.1 (0.99)	(0.9, 1.3)
Co-occurring binary (≥ 3 risks)		21/284	7.3%	(2.8,17.8)	19/225	8.3%	(4.0,16.6)	39/510	7.7%	(3.6,15.9)

NB: Population N will differ for each risk, as participants could skip survey questions if they chose

*MVPA* Moderate to Vigorous Physical Activity, *VPA* Vigorous Physical Activity, *TV* Television, *HDL* High Density Lipoprotein

^a^Weighted mean (standard deviation)

#### Biomarkers

Of the biomarkers measured in the Jakarta sample, reduced HDL cholesterol was the most prevalent, affecting 54.9% (95%CI 46.4 to 63.1) of our sample. Raised triglycerides, raised blood glucose levels (evidence of prediabetes), and raised blood pressure were all more prevalent in males compared to females, while reduced HDL-cholesterol and high waist circumference was higher in females compared to males. Overall, 7.7% (95%CI 3.6 to 15.9) of our subsample had evidence of co-occurring cardiometabolic risks, with a mean of 1.1 (SD = 0.99).

### Socio-demographic differences

**Fig. 2:**
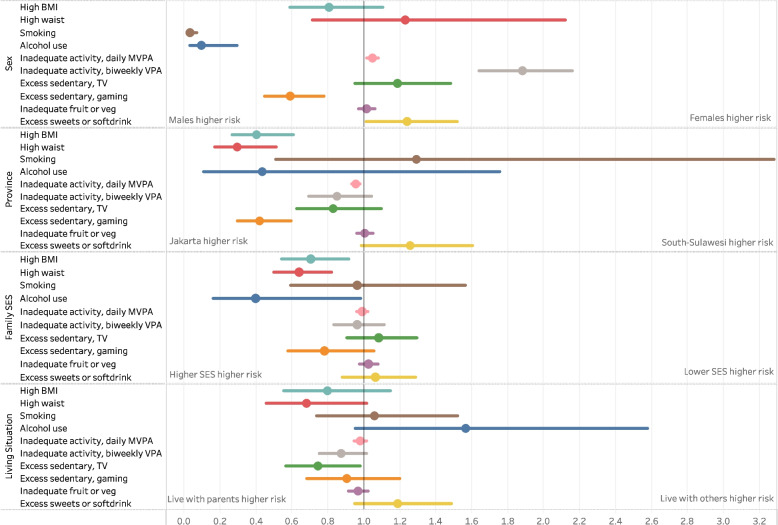
Risk ratios for population weighted prevalence of NCD risk factors, by socio-demographic factors, bars indicate 95%CI’s

Large sex differences were evident across some risks, see Fig. 2, and appendix Table A1. The prevalence of smoking tobacco was significantly higher in males (32.3%) than females (1%), RR 0.03 (95%CI 0.01–0.07). Similarly, prevalence of alcohol use in males was tenfold that of females, RR 0.09 (95%CI 0.03–0.29). Females were at a greater risk of not getting enough vigorous activity than males (65.3% and 34.7% respectively, RR 1.88, 95%CI 1.64–2.16).

Compared to those in South Sulawesi, adolescents in Jakarta had increased prevalence of high BMI (RR 0.40, 95%CI 0.27–0.61), high waist circumference (RR 0.29, 95%CI 0.17–0.51), a lack of daily MVPA (RR 0.95, 95%CI 0.93–0.98), and sedentary behaviour (RR 0.63, 95%CI 0.52–0.76). Adolescents self-identifying as a higher socioeconomic status had significantly greater risk of high BMI (RR 0.70, 95%CI 0.54–0.91), high waist circumference (RR 0.64, 95%CI 0.50–0.82), and alcohol use (RR 0.40, 95%CI 0.16–0.98), compared to their lower SES counterparts. There were no particularly strong associations between living situation (whether adolescents lived with their parents, or with others) and the risk factors.

### Primary Outcomes: Indicators for mental wellbeing

The mean psychological distress score overall was 12.8 (SD = 7). Table 2 shows there was divergence between the sexes with the mean score for females higher than the mean score for males. For general QoL the mean scores were similar between the sexes, however for physical functioning males reported a higher mean score of 81.2 (SD = 15.3), compared to females with a mean of 74.2 (SD = 15.5).

### The relationship between individual NCD risk factors and indicators of wellbeing

Table 4 shows the associations between exposure to NCD risk factors and wellbeing. Overall, exposure to NCD risk factors was associated with lower mean scores in physical functioning and general QoL, and a higher mean score for psychological distress. Psychological distress was significantly associated with eight out of ten risk factors. Physical function QoL was associated with five out of ten risks, and general QoL with four out of ten risks. Excess time spent watching TV did not appear to have a strong relationship with any of the wellbeing indicators. When we examined the associations between exposure to NCD risk factors and wellbeing by sex, we found similar trends across the individual risk factors; lower mean scores in physical functioning and general QoL, and a higher mean score for psychological distress for both males and females. We did find more individual risk factors associated with psychological distress for males than females (see appendix Table A2).

**Table 4:** Wellbeing outcomes by individual and co-occurring NCD risk factors, in adolescents

		Psychological distress (scale: 0–40)		Physical function quality of life (scale: 0–100)		Youth general quality of life (scale: 0–100)	
NCD risks^a^ in Jakarta and South Sulawesi							
		Mean Diff	95% CI	Mean Diff	95% CI	Mean Diff	95% CI
Adiposity	High BMI	1.04*	(0.2, 1.9)	−4.05*	(−6.3, −1.8)	−1.08	(−4.4, 2.2)
	High waist	1.29	(−0.0, 2.6)	−3.65*	(−6.7, −0.6)	−1.19	(−4.8, 2.4)
Substance use	Smoking	1.50*	(0.1, 3.0)	−2.77	(−7.3, 1.8)	−5.46*	(−10.7, −0.3)
	Alcohol use	2.04*	(0.1, 3.9)	−0.54	(−4.8, 3.7)	−3.49	(−9.2, 2.2)
Physical inactivity	Inadequate MVPA	2.35*	(0.6, 4.1)	−5.56*	(−10.1, −1.1)	−12.63*	(−16.7, −8.6)
	Inadequate vigorous PA	0.87*	(0.1, 1.7)	−6.46*	(−8.9, −4.0)	−3.54*	(−6.0, −1.1)
Sedentary	Excess TV	−0.05	(−0.9, 0.8)	−0.29	(−2.3, 1.8)	2.59	(−0.1, 5.2)
	Excess gaming	2.21*	(0.9, 3.5)	−3.04*	(−5.7, −0.4)	0.90	(−2.3, 4.1)
Diet	Inadequate fruit or veg	1.79*	(0.8, 2.8)	−2.83	(−6.1, 0.5)	−5.96*	(−9.6, −2.3)
	Excess sweets/ soft drink	0.92*	(0.1, 1.7)	−0.11	(−2.0, 1.8)	1.00	(−1.3, 3.3)
		Coeff	95% CI	Coeff	95% CI	Coeff	95% CI
Co-occurring NCD risk count^b^		1.40*	(1.0, 1.8)	−2.54*	(−3.7, −1.4)	−1.68*	(−3.1, −0.3)

NB: Each regression model was adjusted for significantly associated covariates: province, sex, family socioeconomic status, and religious affiliation

^a^NCD risks are binary outcomes where the reference category is the absence of the risk factor, as defined in Table 1

^b^Co-occurring risk counts for NCD risks are continuous measures

^*^Indicates *P* value: *p* < 0.05

For the biomarkers, measured in Jakarta only, we found an association between reduced HDL and raised blood sugar, and increased mean psychological distress score (Table 5). As with the full sample, those with high waist circumference had a lower mean Physical function QoL score, and there were no clear associations between biomarkers and general QoL. When we examined the associations between biomarkers and wellbeing by sex, we found an association between reduced HDL and psychological distress in females, and physical functioning and high waist in males (see appendix Table A3).

**Table 5:** Wellbeing outcomes by individual and co-occurring risk biomarkers, in adolescents

	Psychological distress (scale: 0–40)		Physical function quality of life (scale: 0–100)		Youth general quality of life (scale: 0–100)	
Biomarkers^a^ in Jakarta	Mean Diff	95% CI	Mean Diff	95% CI	Mean Diff	95% CI
Reduced HDL	1.18*	(0.1, 2.2)	−1.47	(−5.0, 2.0)	−1.56	(−3.7, 0.6)
Raised triglycerides	−0.55	(−1.9, 0.8)	−0.70	(−5.0, 3.6)	0.43	(−3.9, 4.8)
Raised blood glucose	3.55*	(0.7, 6.4)	−9.68	(−19.7, 0.3)	−0.98	(−8.0, 6.0)
Raised blood pressure	−0.18	(−2.9, 2.5)	1.46	(−2.4, 5.3)	2.94	(−1.5, 7.4)
High waist	1.25	(−0.4, 2.9)	−4.22*	(−7.8, −0.6)	−0.40	(−4.4, 3.6)
	Coeff	95% CI	Coeff	95% CI	Coeff	95% CI
Co-occurring biomarker count^b^	0.51	(−0.3, 1.3)	−1.43*	(−2.4, −0.5)	−0.02	(−1.6, 1.5)

NB: Each regression model was adjusted for significantly associated covariates: sex, family socioeconomic status, and religious affiliation

^a^Biomarkers are binary outcomes where the reference category is the absence of the risk factor, as defined in Table 1

^b^Co-occurring risk counts for biomarkers are continuous measures

^*^ Indicates *P* value: *p* < 0.05

### The relationship between co-occurrence of risks and wellbeing

The association between exposure to co-occurring NCD risks and both psychological distress and quality of life was evident, Table 4. Even when adjusting for significant covariates (province, sex, family socioeconomic status, and religion) those adolescents in our sample exposed to multiple risks had higher mean psychological distress scores. In our regression model, a one unit increase in risk factor count was associated with an increase in the mean psychological distress score by 1.40 (95%CI 1.0 to 1.8). Like the association with psychological distress, exposure to multiple co-occurring risks resulted in the poorer QoL scores, despite adjusting for all significant covariates. A one unit increase in risk factor count was associated with a decrease in the mean score for physical function by −2.54 (95%CI −3.7 to −1.4) and a decrease in the mean score for general QoL by −1.68 (95%CI −3.1 to −0.3). When we analysed the associations by males and females separately, these trends were repeated overall. A one unit increase in risk factor count was associated with an increase in the mean psychological distress score by 1.14 (95%CI 0.5 to 1.8) for males and by 1.74 (95%CI 1.2 to 2.2) for females. Likewise, a one unit increase in risk factor count was associated with a decrease in the mean score for physical function by −2.45 (95%CI −4.1 to-0.8) for males and by −2.82 (95%CI −4.8 to −0.9) for females. The association between co-occurring risks and a reduction in general quality of life score was significant for males only (see appendix Table A2).

Overall, for those with co-occurring risk biomarkers in Jakarta there were no strong associations with any indicators of wellbeing (Table 5). There was some evidence of an association with lower physical function QoL, with a one unit increase in biomarker count being associated with a decrease in mean physical function score of −1.43 (95%CI −2.4 to −0.5). There were some differences between males and females when examined separately. Males had a similar association between decrease in mean physical function score as biomarker count increased, while for females, a one unit increase in biomarker count was associated with an increase in mean psychological distress score of 1.04 (95%CI 0.2 to 1.9), and there was no significant association for physical function QoL (see appendix Table A3).

## Discussion

This study is the first of its kind for Indonesian adolescents. We found evidence of a significant relationship between the major NCD risk factors and current indicators of mental wellbeing in our novel sample. Psychological distress was associated with every risk domain, physical function QoL was associated with adiposity, physical inactivity, and excess sedentary time, and general QoL was associated with substance use, physical inactivity, and diet. Those adolescents with risk factors present in more risk domains had higher mean scores for psychological distress, a scale which has good ability to detect depression and anxiety in this population [18]. Moreover, we saw a relationship between increasing co-occurring risk factors and a reduction in quality of life across two differing scales, physical function and general QoL. This evidence of current diminished wellbeing indicates that intervening on NCD risk factors could improve the mental health and wellbeing for young people now, not just for the benefit of future adult health.

The trends described here between common NCD risks and wellbeing suggest an important relationship between an adolescent’s current wellbeing and their experience of NCD risk, and notably, the number of co-occurring risks they are exposed to. Interestingly, we did not see this pattern replicated in our subsample with multiple cardiometabolic risk biomarkers. This is consistent with another study in young adults which examined several risk biomarkers, including high fasting glucose, triglycerides, low HDL cholesterol, high blood pressure, and high waist circumference and found a low correlation only between waist circumference and psychological distress [32]. These findings could potentially provide some of the context in which to interpret our results. The social and behavioural nature of many of these risks (such as smoking cigarettes, the foods they consume, or participation in physical activity) could be more closely tied to adolescents’ wellbeing than the cardiometabolic condition indicated by their blood lipid profile. That is not to say the biological impacts measured here are not relevant to wellbeing, there are likely other mediating factors. A recent meta-analysis of cohort studies examining the relationship between clustered metabolic risks and depressive symptoms showed consistently significant associations in western countries, but not in Asian countries [33]. Meanwhile, a population-based study of adults with diabetes found that symptoms of depression had a stronger association with the experience of diabetic symptoms than measures of glycaemic control (HbA(1c) levels) [34]. So, while the relationship with co-occurring biomarkers and mental wellbeing may be less clear in our adolescent sample, there is an interesting and novel association between NCD risks and mental wellbeing. Further research is needed to unpack the direction of these associations, but the importance of this finding and the opportunities to prevent ill-health could be substantial.

There have been calls over the past decade to consider the significance of the major NCD risk factors in a public health approach to prevention and control of common mental disorders [35, 36]. These risks are largely modifiable, and while the effect on mental health on the individual level may not be as significant as preventing the major risks for mental disorder, such as adverse childhood experiences or poly-victimisation, the potential impact on wellbeing at the population level (and cumulatively across disease outcomes) should not be overlooked [37, 38]. Results from previous studies have been mixed; systematic reviews by Wu et al. [16] and Hoare et al. [13, 39] found a relationship between several NCD risks and HRQOL, and depressive symptoms, respectively, however the authors questioned the quality and representativeness of the studies reviewed in both cases [16, 39]. Our cross-sectional results indicate that there is an association present between NCD risks and adolescent wellbeing. Future studies into effective interventions to address NCD risks could consider measuring wellbeing in young people as a potential short-term outcome, alongside other positive outcomes such as reducing long-term likelihood of developing an NCD in later life, or reducing the associated disability experienced from potential NCD outcomes if they arise [40]. If there were effective interventions which could be shown to improve both current wellbeing as well as future health outcomes, the immediate impact on wellbeing could be a stronger motivator for adolescents than those actions promising a healthier future. The mixed results from previous studies, in the context of our strong results indicate the need for further research in this field to examine the complex relationships explored here, in this important age group.

Socio-demographic factors at the individual, household, and societal level are often highlighted in the literature as an important part of the underlying mechanisms to explain the close relationship between mental wellbeing, NCDs, and their risk factors [35, 40]. Our large sample enabled us to describe the distribution of key NCD risk factors by relevant socio-demographic factors, and outcomes by sex. Sex differences, while not ubiquitous, were sizeable where present. Males had a notably higher prevalence of substance use, and females had double the risk of inadequate physical activity. This reflects global findings for sex differences in physical inactivity and substance use [41–43]. Adiposity was more prevalent in highly urbanised Jakarta, and likewise amongst those in the sample who self-identified as being of higher-than-average socio-economic status. While this fits with a common global narrative around urbanisation and obesogenic risk factors, recent findings both globally and from Indonesia encourage a more nuanced analysis, with rural BMI increasing faster (despite being lower overall) than in cities in low- and middle-income regions, and people living in Indonesian cities having a dietary pattern which both increases NCD risk (increased consumption of sugary drinks and ultra-processed food) and protects against NCDs (increased consumption of vegetables and fish) [44, 45].

There are some limitations to be addressed. Our study focused on only two of Indonesia’s provinces, which limits generalisability. However, the two provinces selected differ substantially and were chosen for their diversity of setting (urban, rural, peri-urban), population density, adolescent population, and Human Development Index. The cross-sectional nature of this study means that we are unable to address causation, however this study adds to the growing body of research that shows an increasing burden of NCD risk in Indonesian adolescents and to the understanding about the relationship between these risk factors and current wellbeing. The association observed between co-occurring NCD risks and wellbeing, and quality of life is likely bi-directional, and further research is needed to better understand the underlying mechanisms. However, this association indicates that addressing NCD risks now is important for young peoples’ current health and wellbeing and not only for future adult health.

We found evidence of a large proportion of young people in Jakarta and South Sulawesi with co-occurring NCD risks. This is consistent with earlier studies that have suggested an increasing prevalence of co-occurring risks over time [46]. The prevalence of insufficient physical activity using the WHO guideline for adolescents (60 min of daily physical activity of moderate-to-vigorous intensity) was very high (96.1%) in our sample. However, this was consistent with other studies using the same definition of physical inactivity. A 2019 study reported overall prevalence of insufficient physical activity as 86.4% overall in 11–17-year-olds in Indonesia, and a study from 2015 Indonesian Global School Health Survey in 13–16-year-olds found a prevalence of 87.8% [41, 46]. While our estimate was higher by 7–8 percentage points, we had an overall older sample (aged 16 to 18, a known risk for decreasing activity), and our sample was only drawn from two provinces, which could explain some of the elevated risk. Similarly, we had very high levels of inadequate fruit and vegetable consumption within our sample, 91.1%. However, analysis of Indonesia’s RISKESDAS 2018 survey data shows similar levels (over 95%) in 15 to 19-year-olds and 18 to 24-year-olds [47, 48]. Our findings here, and the similarity with other published estimates, indicates that these risks are almost universal. While these are well established risk definitions their highly prevalent nature calls into question their practical utility in identifying those at greater risk than the general population.

The mixed method data collection across a large, representative sample, is a strength of this study. We report on self-report survey items, anthropometrics, and biomarkers for this important population. Many of the other studies with a similar focus are largely reliant on self-report survey data only, and do not have objective measures of height, weight, waist circumference, blood pressure, or blood lipid profiles. Our outcome measures for mental wellbeing and quality of life are measured with sound, validated scales, and our earlier work in culturally verifying and validating the K10 within a subsample of diagnostic interviews (MINI-KID) enables reliable estimates [18, 49]. Another strength of this study is the grounding in qualitative enquiry. Early qualitative work influenced much of the original survey design, ensuring culturally relevant and informed data collection [17, 50]. The analysis presented here was informed by more recent qualitative interviews with stakeholders across Indonesia, working in NCD prevention and adolescent health, and an in-depth understanding of the data needs of those stakeholders [51].

## Conclusion

Our findings show a considerable prevalence of NCD risk factors, including objectively measured biomarkers and anthropometrics, amongst Indonesian adolescents in our sample. Furthermore, we found an association between co-occurring NCD risks and the current wellbeing of adolescents. Compared to previous studies, the increasing prevalence of these individual risks, and the increasing prevalence of individuals with co-occurring risks, highlights an urgent need to intervene in this age group and younger adolescents, to curtail the epidemic of NCDs in Indonesia. Importantly, the association between NCD risks and current wellbeing and quality of life, provides another dimension to benefits that can be attributed to the reduction of NCD risks, alongside the potential to accelerate progress on reducing NCD burden and adds strength to the call for urgent action. In tackling adolescent wellbeing, the additional benefits potentially gained by improving diet, exercise, and reducing substance use should not be overlooked. Likewise, public health interventions aimed at reducing NCDs in adults should consider intervening earlier in the life-course to gain the benefit of improving young people’s current health and wellbeing, alongside the opportunity to reduce the future disease burden and disability. Further systematic research into preventing NCD outcomes, their risks, and understanding the underlying mechanisms, including the impact of social determinants, is still needed across the adolescent life-phase in many countries, including Indonesia.

## Supplementary Information

Supplementary Material 1.

## Data Availability

Reasonable requests for de-identified data will be enabled by the study team within the ethical constraints of the project approvals. Please contact the corresponding author to make a data request via email (karly.cini@mcri.edu.au).
