# Supplementary material for: The relationship between non-communicable disease risk and mental wellbeing in adolescence: a cross-sectional study utilising objective measures in Indonesia
Source: BMC Public Health. 2024 Dec 18;24:3416. doi: 10.1186/s12889-024-20902-1 (PMC11653778; doi:10.1186/s12889-024-20902-1)
Supplement: Supplementary file 1 — Supplementary Material 1. [file 12889_2024_20902_MOESM1_ESM.docx]

# APPENDIX

[SOCIO-DEMOGRAPHIC QUESTIONS 2](#_Toc180711483)

[Religion 2](#_Toc180711484)

[Self-report socio-economic status 2](#_Toc180711485)

[Living situation 2](#_Toc180711486)

[OUTCOMES 2](#_Toc180711487)

[K10 PSYCHOLOGICAL DISTRESS SCALE: 2](#_Toc180711488)

[PEDSQL PHYSICAL FUNCTIONING SCALE: 3](#_Toc180711489)

[YOUTH QUALITY OF LIFE – SHORT FORM: 3](#_Toc180711490)

[SELF-REPORT RISK FACTORS 5](#_Toc180711491)

[SUBSTANCE USE DOMAIN: 5](#_Toc180711492)

[Tobacco Use 5](#_Toc180711493)

[Alcohol Use 5](#_Toc180711494)

[PHYSICAL INACTIVITY DOMAIN: 5](#_Toc180711495)

[Lack of daily moderate to vigorous physical activity (MVPA) 6](#_Toc180711496)

[Lack of vigorous physical activity 6](#_Toc180711497)

[SEDENTARY DOMAIN: 6](#_Toc180711498)

[Excess time spent watching TV 6](#_Toc180711499)

[Excess time spent using the computer or playing video games 7](#_Toc180711500)

[UNHEALTHY DIET DOMAIN: 7](#_Toc180711501)

[Insufficient fruit and vegetables 7](#_Toc180711502)

[Excess sweets and soft drink 7](#_Toc180711503)

[Table A1. Prevalence and risk ratios of NCD risk factors by socio-demographic factors 9](#_Toc180711504)

[Table A2. Wellbeing outcomes by individual and co-occurring NCD risk factors, by sex. 11](#_Toc180711505)

[Table A3. Wellbeing outcomes by individual and co-occurring cardiometabolic risk biomarkers in Jakarta, by sex. 12](#_Toc180711506)

##

## SOCIO-DEMOGRAPHIC QUESTIONS

#### Religion

What religion do you identify with?

| Islam |  |
| --- | --- |
| Christianity |  |
| Buddhism |  |
| Hinduism |  |
| Judaism |  |
| No religion |  |
| Other (please specify):  _____________________ |  |

#### Self-report socio-economic status

How well off do you think your family is?

| Very well off |  |
| --- | --- |
| Quite well off |  |
| Average |  |
| Not very well off |  |
| Not at all well off |  |

#### Living situation

Who do you live with now (please tick all that apply)?

| Mother |  |
| --- | --- |
| Father |  |
| Stepmother (or father’s girlfriend) |  |
| Stepfather (or mother’s girlfriend) |  |
| Grandmother |  |
| Grandfather |  |
| I live in a foster home |  |
| Other (please specify): |  |

## OUTCOMES

### K10 PSYCHOLOGICAL DISTRESS SCALE:

Refer to: Kessler RC, Barker PR, Colpe LJ, Epstein JF, Gfroerer JC, Hiripi E, et al. Screening for serious mental illness in the general population. Archives of general psychiatry. 2003;60(2):184-9.

1. The following questions ask about how you have been feeling during the past 4 weeks. For each question, please tick the response that best describes how often you had this feeling. (please complete each of the questions below)

|  | **None of the time** | **A little of the time** | **Some of the time** | **Most of the time** | **All of the time** |
| --- | --- | --- | --- | --- | --- |
| 1. In the past 4 weeks, about how often did you feel tired out for no good reason? |  |  |  |  |  |
| - 1. In the past 4 weeks, how often did you feel nervous? |  |  |  |  |  |
| - 1. In the past 4 weeks, about how often did you feel so nervous that nothing could calm you down? |  |  |  |  |  |
| - 1. In the past 4 weeks, about how often did you feel hopeless? |  |  |  |  |  |
| - 1. In the past 4 weeks, about how often did you feel restless or fidgety? |  |  |  |  |  |
| - 1. In the past 4 weeks, about how often did you feel so restless you could not sit still? |  |  |  |  |  |
| - 1. In the past 4 weeks, about how often did you feel depressed? |  |  |  |  |  |
| - 1. In the past 4 weeks, about how often did you feel that everything was an effort? |  |  |  |  |  |
| - 1. In the past 4 weeks, about how often did you feel so sad that nothing could cheer you up? |  |  |  |  |  |
| - 1. In the past 4 weeks, about how often did you feel worthless? |  |  |  |  |  |

### PEDSQL PHYSICAL FUNCTIONING SCALE:

Refer to: Varni JW, Seid M, Rode CA. The PedsQL: measurement model for the pediatric quality of life inventory. Medical care. 1999;37(2):126-39.

1. In the past 30 days, how much of a problem has this been for you?

|  | **Never** | **Almost Never** | **Sometimes** | **Often** | **Almost Always** |
| --- | --- | --- | --- | --- | --- |
| - 1. It is hard for me to walk more than one block (100 meters) |  |  |  |  |  |
| - 1. It is hard for me to run |  |  |  |  |  |
| - 1. It is hard for me to do sports activity or exercise |  |  |  |  |  |
| - 1. It is hard for me to lift something heavy |  |  |  |  |  |
| - 1. It is hard for me to take a bath or shower by myself |  |  |  |  |  |
| - 1. It is hard for me to do chores around the house |  |  |  |  |  |
| - 1. I hurt or ache |  |  |  |  |  |
| - 1. I have low energy |  |  |  |  |  |

### YOUTH QUALITY OF LIFE – SHORT FORM:

Refer to: Patrick DL, Edwards TC, Topolski TD. Adolescent quality of life, part II: initial validation of a new instrument. J Adolesc. 2002;25(3):287-300.

1. Following are some statements that you might make about yourself. Please circle the one number on each scale that best describes how closely the statement applies to you IN GENERAL. There are no right or wrong answers, we are only interested in how you feel about your life. (*qol_1 – qol_15*)
2. I am able to do most things as well as I want (please circle one number)

| Not at all | 0 | 1 | 2 | 3 | 4 | 5 | 6 | 7 | 8 | 9 | 10 | Very much |
| --- | --- | --- | --- | --- | --- | --- | --- | --- | --- | --- | --- | --- |

1. I feel good about myself (please circle one number)

| Not at all | 0 | 1 | 2 | 3 | 4 | 5 | 6 | 7 | 8 | 9 | 10 | Very much |
| --- | --- | --- | --- | --- | --- | --- | --- | --- | --- | --- | --- | --- |

1. I feel I am important to others (please circle one number)

| Not at all | 0 | 1 | 2 | 3 | 4 | 5 | 6 | 7 | 8 | 9 | 10 | Very much |
| --- | --- | --- | --- | --- | --- | --- | --- | --- | --- | --- | --- | --- |

1. I am pleased with how I look (please circle one number)

| Not at all | 0 | 1 | 2 | 3 | 4 | 5 | 6 | 7 | 8 | 9 | 10 | Very much |
| --- | --- | --- | --- | --- | --- | --- | --- | --- | --- | --- | --- | --- |

1. I feel understood by my parents or guardians (please circle one number)

| Not at all | 0 | 1 | 2 | 3 | 4 | 5 | 6 | 7 | 8 | 9 | 10 | Very much |
| --- | --- | --- | --- | --- | --- | --- | --- | --- | --- | --- | --- | --- |

1. I feel I am getting along with my parents or guardians (please circle one number)

| Not at all | 0 | 1 | 2 | 3 | 4 | 5 | 6 | 7 | 8 | 9 | 10 | Very much |
| --- | --- | --- | --- | --- | --- | --- | --- | --- | --- | --- | --- | --- |

1. I feel alone in my life (please circle one number)

| Not at all | 0 | 1 | 2 | 3 | 4 | 5 | 6 | 7 | 8 | 9 | 10 | Very much |
| --- | --- | --- | --- | --- | --- | --- | --- | --- | --- | --- | --- | --- |

1. I am happy with the friends I have (please circle one number)

| Not at all | 0 | 1 | 2 | 3 | 4 | 5 | 6 | 7 | 8 | 9 | 10 | Very much |
| --- | --- | --- | --- | --- | --- | --- | --- | --- | --- | --- | --- | --- |

1. I feel I can take part in the same activities as others my age (please circle one number)

| Not at all | 0 | 1 | 2 | 3 | 4 | 5 | 6 | 7 | 8 | 9 | 10 | Very much |
| --- | --- | --- | --- | --- | --- | --- | --- | --- | --- | --- | --- | --- |

1. People my age treat me with respect (please circle one number)

| Not at all | 0 | 1 | 2 | 3 | 4 | 5 | 6 | 7 | 8 | 9 | 10 | Very much |
| --- | --- | --- | --- | --- | --- | --- | --- | --- | --- | --- | --- | --- |

1. I feel my life is full of interesting things to do (please circle one number)

| Not at all | 0 | 1 | 2 | 3 | 4 | 5 | 6 | 7 | 8 | 9 | 10 | Very much |
| --- | --- | --- | --- | --- | --- | --- | --- | --- | --- | --- | --- | --- |

1. I look forward to the future (please circle one number)

| Not at all | 0 | 1 | 2 | 3 | 4 | 5 | 6 | 7 | 8 | 9 | 10 | Very much |
| --- | --- | --- | --- | --- | --- | --- | --- | --- | --- | --- | --- | --- |

1. I feel safe when I am at home (please circle one number)

| Not at all | 0 | 1 | 2 | 3 | 4 | 5 | 6 | 7 | 8 | 9 | 10 | Very much |
| --- | --- | --- | --- | --- | --- | --- | --- | --- | --- | --- | --- | --- |

1. I feel I am getting a good education (please circle one number)

| Not at all | 0 | 1 | 2 | 3 | 4 | 5 | 6 | 7 | 8 | 9 | 10 | Very much |
| --- | --- | --- | --- | --- | --- | --- | --- | --- | --- | --- | --- | --- |

1. I am satisfied with the way my life is now (please circle one number)

| Not at all | 0 | 1 | 2 | 3 | 4 | 5 | 6 | 7 | 8 | 9 | 10 | Very much |
| --- | --- | --- | --- | --- | --- | --- | --- | --- | --- | --- | --- | --- |

## SELF-REPORT RISK FACTORS

### SUBSTANCE USE DOMAIN:

Tobacco Use

Adapted from: WHO and Centers for Disease Control and Prevention. Global Youth Tobacco Survey (GYTS). 1998. Available from: <https://www.who.int/tobacco/surveillance/gyts/en/>.

2 items: Questions relating to current cigarette smoking, and frequency of smoking. Risk was defined as smoking cigarettes at a frequency of weekly or more.

1. Have you ever tried or experimented with cigarette smoking, even one or two puffs?

| Yes |  |  |
| --- | --- | --- |
| No |  |  |

1. (IF YES) How often do you smoke at present?

| Every day |  |
| --- | --- |
| At least once a week, but not every day |  |
| Less than once a week |  |

#### Alcohol Use

Adapted from: Centers for Disease Control and Prevention. Youth Risk Behaviour Surveillance System (YRBSS). 2018 (Available from: https://www.cdc.gov/healthyyouth/data/yrbs/questionnaires.htm.)

2 items: Questions relating to lifetime and current frequency of alcohol use in the past 30 days. Risk was defined as at least one drink of alcohol in the past 30 days.

1. The next questions ask about drinking alcohol. This includes drinking beer, wine, and liquor such as rum, gin, vodka or whiskey. For these questions, drinking alcohol does not include drinking a few sips of wine for religious purposes.

Have you ever tried alcohol?

| Yes |  |
| --- | --- |
| No |  |

1. (IF YES) During the past 30 days, how often did you have at least one drink containing alcohol?

| I did not have one drink in the past 30 days |  |
| --- | --- |
| Once |  |
| Twice |  |
| A few times |  |
| Weekly |  |
| A few days per week |  |
| Daily |  |

### PHYSICAL INACTIVITY DOMAIN:

Dietary intake, physical activity, and sedentary time questions adapted from HSBC; refer to:

- Roberts C, Freeman J, Samdal O, Schnohr CW, de Looze ME, Nic Gabhainn S, et al. The Health Behaviour in School-aged Children (HBSC) study: methodological developments and current tensions. International Journal of Public Health. 2009;54(2):140-50;
- Currie C, Nic Gabhainn S, Godeau E, The International HBSC Network Coordinating Committee. The Health Behaviour in School-aged Children: WHO Collaborative Cross-National (HBSC) Study: origins, concept, history and development 1982–2008. International Journal of Public Health. 2009;54(2):131-9.

#### Lack of daily moderate to vigorous physical activity (MVPA)

1 item: Question on frequency and duration of physical activity over the past week. Equivalent to WHO PA guideline for 11-17-year-olds. Risk was defined if an individual did not get 60 mins of moderate to vigorous physical activity daily.

1. The next question is about physical activity. Some examples are running, brisk walking, biking, dancing, skateboarding, swimming, and badminton. For this next question, add up all the time you spent in physical activity each day.

Over the past 7 days, on how many days were you physically active for a total of at least 60 minutes per day? (please tick)

| Number of days | | | | | | | |
| --- | --- | --- | --- | --- | --- | --- | --- |
| 0 | 1 | 2 | 3 | 4 | 5 | 6 | 7 |
|  |  |  |  |  |  |  |  |

#### Lack of vigorous physical activity

1 item: Question on frequency of vigorous physical activity over the past month. Risk was defined if an individual did vigorous physical activity less than twice per week.

1. How OFTEN do you usually exercise in your free time so much that you get out of breath or sweat? (please tick the one that applies to you)

| Every day |  |
| --- | --- |
| 4 to 6 times/week |  |
| 2 to 3 times/ week |  |
| Once a week |  |
| Once a month |  |
| Less than once/month |  |
| Never |  |

### SEDENTARY DOMAIN:

See above for references, adapted from HBSC.

#### Excess time spent watching TV

1 item: Questions relating to time spent watching TV (on average). Risk was defined as watching TV more than 2hrs per day.

1. On average, how many hours do you watch TV per day? (tick one)

| I do not watch TV |  |
| --- | --- |
| Less than 1 hour per day |  |
| 1 hour per day |  |
| 2 hours per day |  |
| 3 hours per day |  |
| 4 hours per day |  |
| 5 or more hours per day |  |
| I do not have a TV |  |

#### Excess time spent using the computer or playing video games

1 item: Questions relating to time spent using a laptop and playing video games (on average). Risk was defined as gaming/ computer use more than 2hrs per day.

1. On average, how many hours do you play video or computer games or use a computer/laptop? (tick one)

| I do not play video or computer games or use a computer/laptop |  |
| --- | --- |
| Less than 1 hour per day |  |
| 1 hour per day |  |
| 2 hours per day |  |
| 3 hours per day |  |
| 4 hours per day |  |
| 5 or more hours per day |  |
| I do not have a video or computer games or a computer/laptop |  |

### UNHEALTHY DIET DOMAIN:

See above for references, adapted from HBSC.

#### Insufficient fruit and vegetables

2 items: Questions relating to weekly consumption of fruits, and of vegetables. Risk was defined as fruits or vegetables consumed less than daily.

#### Excess sweets and soft drink

2 items: Questions relating to weekly consumption of sweets, and of soft drinks. Risk was defined as sweets or soft drinks consumed at least daily.

1. When you are not fasting, how many times a week do you usually eat or drink? (Please tick one box for each line)

|  | Never | Less than once/  Week | Once a week | 2-4 days/week | 5-6 days/week | Once a day | More than once/day |
| --- | --- | --- | --- | --- | --- | --- | --- |
| - 1. 1.Fruits | □ | □ | □ | □ | □ | □ | □ |
| - 1. 2.Vegetables | □ | □ | □ | □ | □ | □ | □ |
| - 1. 3. Sweets (e.g. candy or chocolate) | □ | □ | □ | □ | □ | □ | □ |
| - 1. 4. Soft drinks (e.g. coke) | □ | □ | □ | □ | □ | □ | □ |

## Table A1. Prevalence and risk ratios of NCD risk factors by socio-demographic factors

|  |  | **SEX** | | **PROVINCE** | | **SES** | | **LIVING** | |
| --- | --- | --- | --- | --- | --- | --- | --- | --- | --- |
| **Domain** | **Individual risk factor** | **Male** | **Female** | **Jakarta** | **South Sulawesi** | **SES Average or higher** | **SES Below average** | **Live with parents or stepparents** | **Live with others (grandparents, friends, others)** |
| **Adiposity** | **High BMI** |  |  |  |  |  |  |  |  |
|  | n/N | 117/596 | 117/735 | 169/680 | 65/651 | 133/633 | 94/637 | 188/1038 | 37/260 |
|  | Prevalence (95%CI) | 19.70 (14.41,26.34) | 15.86 (12.68,19.67) | 24.86 (18.76,32.15) | 9.97 (7.26,13.54) | 20.96 (15.64,27.49) | 14.73 (11.90,18.11) | 18.12 (14.22,22.80) | 14.42 (9.74,20.83) |
|  | Risk Ratio (95%CI) | REFERENCE | 0.80 (0.59,1.10) | REFERENCE | 0.40* (0.27,0.61) | REFERENCE | 0.70* (0.54,0.91) | REFERENCE | 0.80 (0.55,1.15) |
|  | **High waist** |  |  |  |  |  |  |  |  |
|  | n/N | 63/596 | 96/735 | 124/680 | 35/651 | 94/633 | 61/637 | 133/1038 | 23/260 |
|  | Prevalence (95%CI) | 10.58 (6.53,16.70) | 13.00 (9.11,18.22) | 18.21 (12.78,25.27) | 5.35 (3.44,8.21) | 14.91 (11.03,19.86) | 9.52 (6.67,13.40) | 12.81 (9.26,17.46) | 8.70 (5.75,12.95) |
|  | Risk Ratio (95%CI) | REFERENCE | 1.23 (0.71,2.12) | REFERENCE | 0.29* (0.17,0.51) | REFERENCE | 0.64* (0.50,0.82) | REFERENCE | 0.68 (0.46,1.01) |
| **Substance use** | **Smoking** |  |  |  |  |  |  |  |  |
|  | n/N | 141/436 | 6/625 | 71/576 | 77/486 | 75/523 | 69/498 | 113/831 | 29/203 |
|  | Prevalence (95%CI) | 32.31 (23.57,42.48) | 1.01 (0.47,2.14) | 12.25 (5.61,24.68) | 15.81 (8.84,26.67) | 14.30 (7.72,24.96) | 13.74 (8.61,21.24) | 13.65 (8.29,21.65) | 14.43 (9.00,22.34) |
|  | Risk Ratio (95%CI) | REFERENCE | 0.03* (0.01,0.07) | REFERENCE | 1.29 (0.51,3.28) | REFERENCE | 0.96 (0.59,1.56) | REFERENCE | 1.06 (0.74,1.52) |
|  | **Alcohol use** |  |  |  |  |  |  |  |  |
|  | n/N | 71/568 | 8/707 | 57/664 | 23/611 | 56/613 | 22/606 | 55/999 | 21/247 |
|  | Prevalence (95%CI) | 12.57 (4.89,28.69) | 1.19 (0.56,2.50) | 8.59 (2.23,27.88) | 3.72 (2.17,6.31) | 9.11 (2.97,24.73) | 3.61 (1.95,6.58) | 5.49 (2.39,12.08) | 8.59 (2.77,23.63) |
|  | Risk Ratio (95%CI) | REFERENCE | 0.09* (0.03,0.29) | REFERENCE | 0.43 (0.11,1.75) | REFERENCE | 0.40* (0.16,0.98) | REFERENCE | 1.56 (0.95,2.58) |
| **Physical inactivity** | **Inadequate MVPA** |  |  |  |  |  |  |  |  |
|  | n/N | 547/595 | 707/735 | 656/680 | 597/650 | 600/633 | 598/637 | 982/1037 | 241/260 |
|  | Prevalence (95%CI) | 91.88 (89.36,93.85) | 96.15 (94.79,97.16) | 96.42 (95.68,97.04) | 91.95 (89.59,93.82) | 94.87 (93.66,95.86) | 93.85 (90.97,95.85) | 94.64 (93.20,95.79) | 92.59 (89.01,95.07) |
|  | Risk Ratio (95%CI) | REFERENCE | 1.05* (1.01,1.08) | REFERENCE | 0.95* (0.93,0.98) | REFERENCE | 0.99 (0.96,1.02) | REFERENCE | 0.98 (0.94,1.01) |
|  | **Inadequate VPA** |  |  |  |  |  |  |  |  |
|  | n/N | 207/595 | 480/735 | 379/680 | 307/650 | 333/633 | 323/637 | 549/1037 | 120/260 |
|  | Prevalence (95%CI) | 34.72 (30.21,39.53) | 65.31 (59.82,70.41) | 55.74 (47.89,63.31) | 47.31 (40.33,54.40) | 52.71 (45.39,59.92) | 50.68 (45.17,56.17) | 52.97 (48.02,57.86) | 46.20 (37.52,55.11) |
|  | Risk Ratio (95%CI) | REFERENCE | 1.88* (1.64,2.16) | REFERENCE | 0.85 (0.69,1.04) | REFERENCE | 0.96 (0.83,1.11) | REFERENCE | 0.87 (0.75,1.01) |
| **Sedentary** | **Excess sedentary, TV** |  |  |  |  |  |  |  |  |
|  | n/N | 179/590 | 261/728 | 247/677 | 194/642 | 201/629 | 218/629 | 365/1031 | 67/254 |
|  | Prevalence (95%CI) | 30.30 (24.73,36.50) | 35.91 (30.19,42.07) | 36.46 (29.16,44.44) | 30.17 (24.90,36.03) | 32.02 (26.13,38.55) | 34.62 (29.65,39.96) | 35.44 (30.52,40.69) | 26.33 (19.80,34.09) |
|  | Risk Ratio (95%CI) | REFERENCE | 1.19 (0.95,1.48) | REFERENCE | 0.83 (0.63,1.10) | REFERENCE | 1.08 (0.90,1.29) | REFERENCE | 0.74* (0.56,0.98) |
|  | **Excess sedentary, gaming** |  |  |  |  |  |  |  |  |
|  | n/N | 210/546 | 150/662 | 256/617 | 103/592 | 199/594 | 148/567 | 289/944 | 65/236 |
|  | Prevalence (95%CI) | 38.36 (31.21,46.06) | 22.59 (17.87,28.12) | 41.54 (32.59,51.08) | 17.38 (13.20,22.54) | 33.41 (26.02,41.71) | 26.03 (20.70,32.18) | 30.59 (25.55,36.14) | 27.63 (19.64,37.37) |
|  | Risk Ratio (95%CI) | REFERENCE | 0.59* (0.45,0.78) | REFERENCE | 0.42* (0.29,0.59) | REFERENCE | 0.78 (0.58,1.05) | REFERENCE | 0.90 (0.68,1.20) |
| **Diet** | **Lack of fruit or veg** |  |  |  |  |  |  |  |  |
|  | n/N | 536/596 | 670/735 | 615/680 | 590/651 | 566/633 | 583/637 | 947/1038 | 229/260 |
|  | Prevalence (95%CI) | 89.90 (86.17,92.71) | 91.13 (88.28,93.33) | 90.43 (87.47,92.75) | 90.73 (87.11,93.41) | 89.27 (85.72,92.02) | 91.44 (87.85,94.04) | 91.28 (89.16,93.02) | 88.17 (82.12,92.37) |
|  | Risk Ratio (95%CI) | REFERENCE | 1.01 (0.97,1.06) | REFERENCE | REFERENCE (0.96,1.05) | REFERENCE | 1.02 (0.97,1.08) | REFERENCE | 0.97 (0.91,1.02) |
|  | **Excess sweets or soft drink** |  |  |  |  |  |  |  |  |
|  | n/N | 152/596 | 232/735 | 175/680 | 210/651 | 181/633 | 193/637 | 287/1038 | 85/260 |
|  | Prevalence (95%CI) | 25.53 (21.43,30.11) | 31.63 (27.50,36.07) | 25.68 (21.16,30.79) | 32.26 (27.45,37.48) | 28.55 (24.82,32.61) | 30.35 (25.48,35.70) | 27.66 (24.41,31.17) | 32.82 (25.65,40.90) |
|  | Risk Ratio (95%CI) | REFERENCE | 1.24* (1.01,1.52) | REFERENCE | 1.26 (0.98,1.60) | REFERENCE | 1.06 (0.88,1.29) | REFERENCE | 1.19 (0.95,1.49) |

*Indicates P value: p < 0.05. NB: Poisson regression with robust error variance was used to calculate relative risk ratios, 95% confidence intervals, and p value, for each risk factor by each socio-demographic factor. No other covariates were included in the model.

##

## Table A2. Wellbeing outcomes by individual and co-occurring NCD risk factors, by sex.

|  | FEMALES | | | | | | MALES | | | | | |
| --- | --- | --- | --- | --- | --- | --- | --- | --- | --- | --- | --- | --- |
|  | Psychological distress  (scale: 0-40) | | Physical function quality of life  (scale: 0-100) | | Youth general quality of life  (scale: 0-100) | | Psychological distress  (scale: 0-40) | | Physical function quality of life  (scale: 0-100) | | Youth general quality of life  (scale: 0-100) | |
|  | Mean Diff. | 95% CI | Mean Diff. | 95% CI | Mean Diff. | 95% CI | Mean Diff. | 95% CI | Mean Diff. | 95% CI | Mean Diff. | 95% CI |
| High BMI | 0.70 | (-0.8, 2.1) | -4.22* | (-7.6,-0.8) | -0.08 | (-4.6, 4.5) | 1.42* | (0.5, 2.3) | -4.13* | (-6.9,-1.3) | -1.99 | (-6.3, 2.4) |
| High waist | 1.48 | (-0.4, 3.3) | -3.28 | (-6.9, 0.4) | -2.29 | (-8.0, 3.5) | 0.93 | (-1.2, 3.1) | -4.35* | (-8.4,-0.4) | 0.80 | (-4.3, 5.9) |
| Smoking | 2.96 | (-2.7, 8.6) | -15.09 | (-34.0, 3.8) | -11.97 | (-32.3, 8.4) | 1.36 | (-0.4, 3.1) | -2.15 | (-6.7, 2.4) | -4.74* | (-9.3,-0.2) |
| Alcohol use | 3.56 | (-0.8, 7.9) | -8.06 | (-21.9, 5.8) | -8.94 | (-20.3, 2.4) | 2.07* | (0.3, 3.8) | 0.06 | (-3.1, 3.3) | -2.52 | (-9.1, 4.1) |
| Inadequate MVPA | 0.76 | (-2.6, 4.1) | -4.22 | (-11.7, 3.2) | -13.64* | (-22.3,-5.0) | 3.43* | (1.9, 5.0) | -6.48* | (-11.8,-1.2) | -11.97* | (-16.2,-7.7) |
| Inadequate vigorous PA | 0.18 | (-1.1, 1.5) | -4.24* | (-6.9,-1.6) | -2.88 | (-6.6, 0.8) | 1.78* | (0.3, 3.3) | -9.29* | (-12.9,-5.7) | -4.53* | (-7.9,-1.2) |
| Excess TV | -0.05 | (-1.2, 1.1) | 0.27 | (-2.2, 2.7) | 2.86 | (-0.4, 6.1) | -0.04 | (-1.5, 1.5) | -1.03 | (-4.0, 1.9) | 2.09 | (-0.9, 5.1) |
| Excess gaming | 3.65* | (2.2, 5.1) | -4.33* | (-7.3,-1.3) | -0.86 | (-5.2, 3.4) | 0.83 | (-0.9, 2.6) | -1.94 | (-5.4, 1.6) | 2.48 | (-0.8, 5.7) |
| Inadequate fruit or veg | 1.73 | (-0.1, 3.5) | -2.19 | (-7.1, 2.7) | -4.96* | (-9.5,-0.4) | 1.85* | (0.1, 3.6) | -3.44 | (-8.9, 2.0) | -7.20* | (-12.5,-1.9) |
| Excess sweets/ soft drink | 1.33* | (0.0, 2.6) | -0.97 | (-3.7, 1.7) | -0.90 | (-3.8, 2.0) | 0.38 | (-0.9, 1.7) | 1.25 | (-2.3, 4.8) | 3.51* | (0.3, 6.7) |
|  |  |  |  |  |  |  |  |  |  |  |  |  |
|  | Coeff | 95% CI | Coeff | 95% CI | Coeff | 95% CI | Coeff | 95% CI | Coeff | 95% CI | Coeff | 95% CI |
| Co-occurring NCD risk count^ | 1.74* | (1.2, 2.2) | -2.82* | (-4.8,-0.9) | -0.85 | (-3.6, 1.9) | 1.14* | (0.5, 1.8) | -2.45* | (-4.1,-0.8) | -2.32* | (-3.8,-0.8) |

NB: Each regression model was adjusted for significantly associated covariates: province, family socioeconomic status, and religious affiliation. #NCD risks are binary outcomes where the reference category is the absence of the risk factor, as defined in Table 1. ^Co-occurring risk counts for NCD risks are continuous measures. * Indicates P value: p < 0.05.

##

## Table A3. Wellbeing outcomes by individual and co-occurring cardiometabolic risk biomarkers in Jakarta, by sex.

|  | FEMALES | | | | | | MALES | | | | | |
| --- | --- | --- | --- | --- | --- | --- | --- | --- | --- | --- | --- | --- |
|  | Psychological distress  (scale: 0-40) | | Physical function quality of life  (scale: 0-100) | | Youth general quality of life  (scale: 0-100) | | Psychological distress  (scale: 0-40) | | Physical function quality of life  (scale: 0-100) | | Youth general quality of life  (scale: 0-100) | |
| Biomarkers^#^ in Jakarta | Mean Diff. | 95% CI | Mean Diff. | 95% CI | Mean Diff. | 95% CI | Mean Diff. | 95% CI | Mean Diff. | 95% CI | Mean Diff. | 95% CI |
| Reduced HDL | 2.03* | (0.6, 3.4) | -1.29 | (-6.9, 4.3) | -0.81 | (-2.6, 1.0) | 0.14 | (-1.4, 1.7) | -1.86 | (-5.2, 1.5) | -2.69 | (-6.3, 1.0) |
| Raised triglycerides | 0.27 | (-2.0, 2.6) | 1.00 | (-2.7, 4.7) | -0.10 | (-6.2, 6.0) | -1.22 | (-3.5, 1.0) | -2.36 | (-10.2, 5.4) | 0.82 | (-3.5, 5.1) |
| Raised blood glucose | 1.42 | (-3.9, 6.7) | -0.51 | (-7.7, 6.7) | -4.99 | (-14.0, 4.0) | 5.50 | (-0.5,11.5) | -17.29 | (-35.2, 0.6) | 2.67 | (-8.0,13.3) |
| Raised blood pressure | 0.52 | (-5.2, 6.2) | 3.40 | (-2.5, 9.3) | 2.70 | (-5.7,11.1) | -0.60 | (-2.6, 1.4) | 0.18 | (-4.9, 5.3) | 3.11 | (-3.1, 9.3) |
| High waist | 1.86 | (-0.5, 4.2) | -3.83 | (-8.7, 1.0) | -0.93 | (-8.0, 6.2) | 0.29 | (-2.6, 3.2) | -4.85* | (-9.5,-0.2) | 0.38 | (-5.9, 6.6) |
|  |  |  |  |  |  |  |  |  |  |  |  |  |
|  | Coeff. | 95% CI | Coeff. | 95% CI | Coeff. | 95% CI | Coeff. | 95% CI | Coeff. | 95% CI | Coeff. | 95% CI |
| Co-occurring biomarker count^ | 1.04* | (0.2, 1.9) | -0.58 | (-1.9, 0.7) | -0.42 | (-3.0, 2.1) | -0.02 | (-1.1, 1.1) | -2.27* | (-3.9,-0.6) | 0.35 | (-1.3, 2.0) |

NB: Each regression model was adjusted for significantly associated covariates: family socioeconomic status and religious affiliation. # Biomarkers are binary outcomes where the reference category is the absence of the risk factor, as defined in Table 1. ^Co-occurring risk counts for biomarkers are continuous measures. * Indicates P value: p < 0.05.
